# Generative Adversarial Network-Based Scheme for Diagnosing Faults in Cyber-Physical Power Systems

**DOI:** 10.3390/s21155173

**Published:** 2021-07-30

**Authors:** Hossein Hassani, Roozbeh Razavi-Far, Mehrdad Saif, Vasile Palade

**Affiliations:** 1 Department of Electrical and Computer Engineering, University of Windsor, Windsor, ON N9B 3P4, Canada; roozbeh@uwindsor.ca (R.R.-F.); msaif@uwindsor.ca (M.S.); 2 School of Computer Science, University of Windsor, Windsor, ON N9B 3P4, Canada; 3 Center for Data Science, Coventry University, Coventry CV1 5FB, UK; vasile.palade@coventry.ac.uk

**Keywords:** generative adversarial networks, feature selection, fault diagnosis, cyber-physical power systems

## Abstract

This paper presents a novel diagnostic framework for distributed power systems that is based on using generative adversarial networks for generating artificial knockoffs in the power grid. The proposed framework makes use of the raw data measurements including voltage, frequency, and phase-angle that are collected from each bus in the cyber-physical power systems. The collected measurements are firstly fed into a feature selection module, where multiple state-of-the-art techniques have been used to extract the most informative features from the initial set of available features. The selected features are inputs to a knockoff generation module, where the generative adversarial networks are employed to generate the corresponding knockoffs of the selected features. The generated knockoffs are then fed into a classification module, in which two different classification models are used for the sake of fault diagnosis. Multiple experiments have been designed to investigate the effect of noise, fault resistance value, and sampling rate on the performance of the proposed framework. The effectiveness of the proposed framework is validated through a comprehensive study on the IEEE 118-bus system.

## 1. Introduction

Complex cyber-physical power systems contain a numerous number of elements such as generation units, bus bars, transmission lines, and loads, which are protected by circuit breakers and protective relays. When a fault happens in an element of the system, a large amount of alarms can potentially be generated by means of the protecting devices to be sent to the supervisory control and data acquisition (SCADA) system [1]. However, it is often difficult to manage the received alarms by means of the SCADA for the sake of fault diagnosis in cyber-physical power systems. Furthermore, the large amount of data measurements collected by means of the sparse measuring devices such as phasor measuring units (PMUs) in SCADA systems makes it even more challenging for the successful implementation of diagnostic frameworks for cyber-physical power systems [2]. Therefore, it is of paramount importance to develop an efficient diagnostic method that can cope with the large amount of data [3].

Data-driven methods have been widely used for fault diagnosis [4]. One of the major challenges in the design of data-driven diagnostic frameworks goes back to the extraction of the most informative features from the numerous number of collected features by means of the SCADA system [5]. This issue, however, can be addressed by resorting to feature selection techniques [6]. Feature selection could be refer to as the process of selecting an informative and relevant subset of the original features [7]. These techniques can generally be divided into three major categories including filters, wrappers, and embedded techniques [8]. Filters makes use of the developed tools for measuring the mutual information, distances, dependencies, and consistencies in order to extract a set of reliable features [9]. Wrappers, however, are generally constructed based on a classification model and take the classification accuracy as a measure in extraction of a subset of features that leads to the best classification accuracy [10]. In embedded techniques, the learning and selection processes are combined and in contrast to the filter or wrapper techniques, these two processes cannot be performed separately [11]. Other than feature selection, developing a diagnostic system that is free of the type of the data distribution is also important for the generalization purposes. To this end, generative adversarial networks (GANs) have been extensively used in the design of data-driven diagnostic frameworks due to their capability of generating the true data distribution from random distributions [12].

GANs are known for their capability in dealing with unbalanced classification by generating realistic looking data samples. They have also found application in data augmentation based on the artificial data which are very similar to the original data samples. Thanks to their advantages, GANs have been widely used in many applications including fault diagnosis [13]. For instance, an auxiliary classifier GAN (ACGAN) has been proposed in [14] to construct an augmentation mechanism for the sake of fault diagnosis. A deep GAN model has been proposed in [15] to deal with the imbalance data used for fault diagnosis. In [16], the authors have used the infrared thermography and infrared image processing in order to generate a useful set of features to evaluate the working condition of angle grinders through a method called BCAoMID-F. The implementation of GANs for self-supervised [17], semi-supervised [18], and unsupervised [12] fault diagnosis schemes have also been extensively studied. In [19], the authors adapt a framework to generate knockoffs, which are random variables that mimic the correlation structure within the existing set of variables in a way that provides a mechanism for the accurate control of the false discovery rate. Even though this technique shows encouraging results, however, it only adapts to the Gaussian distributions. Thanks to the promising results of GANs, the results of this work has been recently extended to a method called knockoffGAN (kGAN), in which the knockoffs are generated without any assumption on the distribution of variables [20]. It is also proposed that by resorting to statistical measures based on the attained coefficients through the Lasso regression, one can employ the feature selection task based on the generated knockoffs. However, a poorly chosen statistic can yield to unreliable results. Therefore, this work aims to propose a diagnostic model based on the knockoffs to benefit from their advantages, while ensures a reliable feature selection for the sake of fault diagnosis.

This work puts forward a novel framework for fault diagnosis in cyber-physical power systems. In contrast to the model-based techniques that rely on the explicit model of the system [21], the proposed framework in this work is data-driven and benefits from the generated knockoffs by means of the kGAN. We consider three different modules in the proposed framework to deal with the large amount of data collected from different spots in the network of the system. Specifically, it is proposed to collect voltage, frequency, and phase-angle features from each bus in the system. After normalization, we make use of a feature selection module in order to construct a subset of the most informative features from the original set of features. In this regard, we make use of the well-known techniques including infinite feature selection (InfFS) [22], mutual information feature selection (MutInfFS) [23], minimum redundancy maximum relevance feature selection (mRMR) [24], and relief feature selection (Relief) [25]. We then run the kGAN technique in the next module of the proposed diagnostic framework, where the selected features from the previous module are used as basis for the generation of knockoffs. The generated knockoffs of the selected features are then fed into the next module, that is the classification module, where two different classification models including k-nearest neighbour (kNN) and support vector machine (SVM) are used to diagnose different types of faults. The main contribution of this work relies on the design of a novel framework that involves multiple modules including feature selection, kGAN, and classification. This is proposed to benefit the most from the advantages of the generative adversarial networks in the extraction of knockoffs. The generated knockoffs are free of the distribution of data and can be generated in a way that controls the false discovery rate of the selected variables. Compared with the scenario, in which no knockoffs are generated from the selected features, the attained results denote the superiority of the proposed framework for classification tasks. We implement three different types of faults on the IEEE 118-bus system and investigate the effect of noise, fault resistance value, and sampling rate on the performance of the proposed framework through a very comprehensive analysis of the attained results.

The rest of this paper is organized as follows. We review the literature of GANs in Section 2. The generation of knockoffs is presented in Section 3. Simulation results and analysis of the attained results are represented in Section 4 and concluding remarks are given in Section 5.

## 2. Literature Review

It is well-studied that GANs consist of two models called generator and discriminator, which are typically implemented by neural networks. The generator model aims to learn the distribution of true examples in order to generate new data samples. The discriminator model, however, aims to discriminate the generated data examples by means of the generator from the true data examples [26]. GANs are constructed based on the generative algorithms, which are a category of machine learning algorithms alongside the discriminative algorithms. The generative algorithms make use of a fully probabilistic model of the observed data and can be categorized into two classes including explicit density model and implicit density model. The former model is based on the distribution of data and tries to train the model either based on the true examples of the distribution or by fitting the distribution parameters. Techniques based on the maximum likelihood estimation, approximate inference [27], and Markov chain [28] are used in training of the explicit models. The implicit models, however, do not rely on the direct estimation or fitting of the distribution parameters. Without any explicit hypothesis, these models generate data samples from a distribution to modify the existing model. The training is typically based on the ancestral sampling [29].

In this regard, different representative variants of GANs have been recently developed for different applications. For instance, InfoGAN [30] in contrast to the typical GAN that makes use of a single unconstructed noise signal, decomposes the noise signal into two parts and tries to derive a lower bound of the mutual information objective for an efficient optimization. Some variants of the InfoGAN including causal InfoGAN [31] and semi-supervised InfoGAN (ss-InfoGAN) [32] have been recently developed. GANs are also extended to the case, in which some extra conditions are assigned to the generator and discriminator models. This model is called conditional GAN (cGAN) [33] and can generate data samples that are conditioned on the class labels [34,35]. For image-to-image translation tasks, where the aim is to learn a mapping from an input image to an output image, cycle-consistent GANs (CycleGAN) have been developed to deal with the issue of unpaired data samples [36]. DualGAN [37] has the same structure as that of the CycleGAN; however, its loss function is supported by the Wasserstein GAN (WGAN) [38]. In contrast to the original GAN, in which the discriminator is used for a binary classification task, the discriminator in WGAN is applied to a regression task in order to estimate the Wasserstein distance. This idea, however, requires the discriminator to be K-Lipschitz constrained. In [39], a method called Wasserstein-divergence (W-div) is proposed to relax the WGAN Lipschitz constraints, where it was then used in WGAN-div to approximate the W-div based on an optimization scheme. Same as the WGAN, loss sensitive GAN (LS-GAN) has also Lipschitz constraints, where the given distribution is assumed to belong to a set of Lipschitz densities with a compact support [40]. These variant models of GANs are trained based on different training structures.

The original GAN is developed based on the multilayer perceptron (MLP). Specifically, the generator and discriminator are MLP models, which can only be used for small-sized datasets and have no good generalization capability to deal with complex images [41]. Laplacian GAN (LAPGAN) [42] has been proposed for higher resolution images and makes use of a cascade of convolutional neural networks (CNN) in a Laplacian framework. In the framework of general GAN model, SinGAN [43] and InGAN [44] have also been proposed to learn a generative model based on a single natural image. The next structure is deep convolutional GAN (DCGAN), where in contrast to the original GAN that makes use of the MLP models, are based on the deep convolutional neural networks (DCNNs) [45]. Progressive GAN (PGGAN) [46] is another category of GAN models, in which the progressive neural networks are used in order to grow the generator and discriminator models progressively. Self-attention GAN (SAGAN) [47] is also another developed structure that utilizes the spectral normalization for generator and discriminator models so as to improve the training dynamics. BigGAN [48] is a recently-developed structure which is similar to the SAGAN, however, it is more scalable. Furthermore, StyleGAN [49] is known for its high-quality generator model in generation of face images. Other structures based on the autoencoders [50], encoders [51], multi-discriminator learning [52], multi-generator learning [53], and multi-GAN learning [54] have also been recently developed for GAN models.

## 3. Knockoff Generation

**Figure 1:**
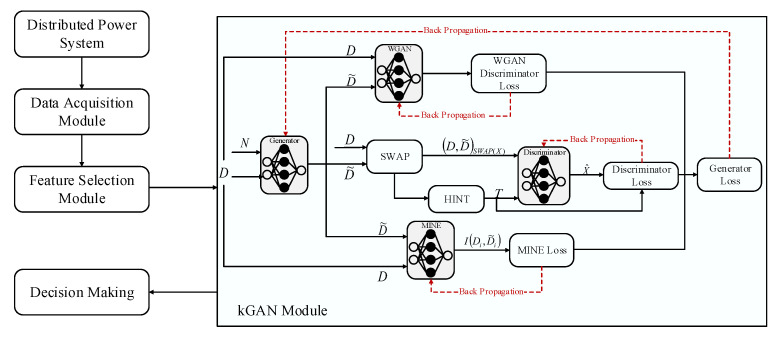
The general framework of the proposed diagnostic method.

The general framework of the proposed method has been illustrated in Figure 1. As it can be observed from this figure, the proposed framework contains multiple modules including data acquisition, feature selection, kGAN, and decision making. We make use of sparse data measuring devices in order to collect voltage, frequency, and phase-angle measurements form each bus in the distributed power system. The collected data measurements are then fed into a feature selection module, in which multiple state-of-the-art techniques including InfFS, Relief, MutInfFS, and mRMR have been implemented in order to extract the most informative features from the original set of features. The extracted features are then fed into the kGAN module, where the selected features are used as input and a corresponding set of random variables called knockoffs are outputs of the module. The generated knockoffs are then fed into the decision making module, where the kNN and SVM classification models have been used in order to diagnose different types of faults.

Assume that the set of features is denoted by D and its dimension is *d*. Suppose that the set of labels is denoted by C and $D=\{D_{1},\ldots ,D_{d}\}$ and *C* are random variables. Then, the concept of a null set can be defined as follows [55].

**Definition** **1.** *A variable $D_{j}$ is null if and only if C is independent of $D_{j}$ conditional on $\left\{D_{j}:i\neq j\right\}$.*

The set of all null variables is shown by K. In order to select the set of most informative features while controlling the false discovery rate, suppose that the set of selected features is denoted by $\hat{X}\subset \left\{1,\ldots ,d\right\}$. The false discovery rate can then be defined as follows:

(1) $$\begin{array}{c} FDR=E\left[\frac{|\hat{X}\cap K|}{|\hat{X}|}\right]. \end{array}$$

Based on the given notations, the definition of the knockoffs can be given as follows [55].

**Definition** **2.** *A knockoff for the variable D is a random variable denoted by $\tilde{D}\in D$ that satisfies the following constraints:*

(2) $$\begin{array}{cc} \left(D,\tilde{D}\right) & \overset{d}{=}{\left(D,\tilde{D}\right)}_{swapX} \end{array}$$

(3) $$\begin{array}{cc} \tilde{D} & ⊥C|D \end{array}$$

*where X∈{1,…,d} and ${\left(.,.\right)}_{swap(X)}$ is used to show the vector that can be obtained by swapping the ith component with the (i+d)th component and $\overset{d}{=}$ denotes the equality on distribution.*

In order to make use of the generated knockoffs for the sake of feature selection, it is required to define a feature statistic $F_{j}$ that only relies on D, $\tilde{D}$, and C. This statistic is defined as $F_{j}=f_{j}\left((D,\tilde{D}),C\right)$ for $f_{j}\in R$. The $f_{j}$ function is required to satisfy the following constraint:

(4) $$\begin{array}{c} F_{j}\left({\left[D,\tilde{D}\right]}_{swap(X)},C\right)=\{\begin{array}{cc} -f_{j}\left(\left[D,\tilde{D}\right],C\right), & j\notin X\\ f_{j}\left(\left[D,\tilde{D}\right],C\right), & \mathrm{otherwise}. \end{array} \end{array}$$

In order to utilize the above statistic, one way is to resort to the LASSO coefficients in order to regress on the augmented set of knockoffs-feature. Denoting the LASSO coefficients by $w_{1},\ldots ,w_{2d}$, one can define the LASSO coefficient difference as follows:

(5) $$\begin{array}{c} F_{j}=|w_{j}\left|-\right|w_{j+d}|. \end{array}$$

Then, based on the given statistic and the definition of knockoffs, the following theorem can be given for the sake of feature selection [55].

**Theorem** **1.** *Suppose that q∈[0,1]. Given the statistics $F_{1},\ldots ,F_{d}$, define:*

(6) $$\begin{array}{c} \tau =\min\left\{t>0:\frac{1+|\{j:F_{j}\leq -t\}|}{|j:F_{j}\geq t|}\leq q\right\}. \end{array}$$

*Then, the selection of variables $\hat{X}=\left\{j:F_{j}\geq \tau \right\}$ will lead to the control of false discovery rate at level q.*

In order to satisfy the given constraints in (2), a modified GAN model, called kGAN, has been used to generate knockoffs without any assumption on the distribution of data. The kGAN module has been illustrated in Figure 1.

As it can be observed from this figure, the kGAN module contains a generator network, denoted by G, that is a function that satisfies $G\left(.,.,\xi \right):D\times {\left[0,1\right]}^{c}\to D$, where its parameters are shown by ξ and takes a random realization of D and random noise $n∼U({\left[0,1\right]}^{c})$ as input and outputs the set of knockoffs $\tilde{D}$.

The discriminator network is designed so as to deal with the given constraint in (2). In this regard, a discriminator network is defined to have a loss which is minimized only for distributions that satisfy the condition given in Equation (2). To this end, the discriminator is denoted by S, which is a function satisfying $S\left(.,\psi \right):D\times D\to {\left[0,1\right]}^{d}$, and takes the swapped sample-knockoff pair ${\left(D,\tilde{D}\right)}_{swap(X)}$ and its output is a vector in ${\left[0,1\right]}^{d}$, where the *i*th component of the output is denoted by $S{\left((D,\tilde{D})\right)}_{swap(X)}$ and denotes the probability of i∈X. To this end, the loss of the discriminator can be given as follows:

(7) $$\begin{array}{c} L_{S}=\sum_{X\in {\left\{0,1\right\}}^{d}}E_{D}\left[E_{\tilde{D}}\left[X.\log{\left(S\left(D,\tilde{D}\right)\right)}_{swap(X)}+\left(1-X\right).\log\left(1-S\left({\left(D,\tilde{D}\right)}_{swap(X)}\right)\right)\right]\right], \end{array}$$

where ‘.’ denotes the dot product. In order to deal with the computational complexity of this loss function, it is suggested to utilize the stochastic gradient descent algorithm for minibatches of *X* that are uniformly sampled. Furthermore, a hint vector T is introduced, which is a random variable to be passed into the discriminator. The introduction of the hint vector involves the sampling of a multivariate Bernoulli random variable B that takes the value of 1 with the probability of 0.9. Then, given $T_{i}=B_{i}$ in case that $B_{i}=1$ and $T_{i}=0.5$ if $B_{i}=0$, the discriminator will then aim to predict only values of *X* for which $B_{i}=0$. To this end, the final loss of the discriminator will be of the following form:

(8) $$\begin{array}{cc} L_{S}=\sum_{X\in {\left\{0,1\right\}}^{d}}E_{D} & [E_{\tilde{D}}[E_{T}[X⊙\left(1-B\right).\log\left(S\left({\left(D,\tilde{D}\right)}_{swap(X)},T\right)\right)+\\ \; & \left(1-T\right)⊙\left(1-B\right).\log(1-S\left({\left(D,\tilde{D}\right)}_{swap(X)},T\right),T)]], \end{array}$$

where ⊙ is the element-wise product.

In order to make the discriminator algorithm more stable, a regularization term of the form of WGAN, denoted by *f* is added to loss function (8). Therefore, the general loss of the discriminator will be of the following form:

(9) $$\begin{array}{c} L_{F}=E\left[f\left(D\right)-f\left(\tilde{D}\right)-\eta (∥{▽}_{\hat{D}f\left(\hat{D}\right)}{{∥}_{2}-1)}^{2}\right], \end{array}$$

where $\hat{D}=\epsilon D+\left(1-\epsilon \right)\tilde{D}$ with ϵ∼U[0,1] and η is parameter to be tuned.

Finally, in order to generate knockoffs that are as independent as possible of the original features, the mutual information neural estimation (MINE) [56] is used to minimize the mutual information between the set of features and their corresponding knockoffs. In this regard, the mutual information between each pair of the feature and knockoff is estimated by means of *d* neural networks, denoted by $N^{1},\ldots ,N^{d}$ with the set of parameters $\theta_{1},\ldots ,\theta_{d}$. By considering a trade-off parameter λ, the following loss of estimation is added to the loss of the generator:

(10) $$\begin{array}{c} L_{P}=\sum_{j=1}^{d}\left(\sum_{i=1}^{n}\left(N_{\theta^{j}}^{j}\left(D_{j}^{\left(i\right)},{\tilde{D}}_{j}^{\left(i\right)}\right)\right)-\log\left(\sum_{i=1}^{n}\exp\left(N_{\theta_{j}}^{j}\left(D_{j}^{k(i)},{\tilde{D}}_{j}^{\left(i\right)}\right)\right)\right)\right), \end{array}$$

in which *k* is supposed to be a permutation of ${\left[n\right]}^{2}$ and superscript (i) is used to demonstrate the *i*th sample. Based on the discussion in this section, the general loss of the proposed method is defined as follows:

(11) $$\begin{array}{c} \min_{G}\left(\max_{S}\left(L_{S}\right)+\lambda \max_{P}\left(L_{P}\right)+\mu \max_{f}\left(L_{f}\right)\right), \end{array}$$

where μ is a parameter to be tuned.

## 4. Simulation Results

In this section, we firstly introduce the IEEE 118-bus power system, and, then, we discuss the types of faults and the generated datasets, and finally, we present the results of the proposed diagnostic framework.

**Figure 2:**
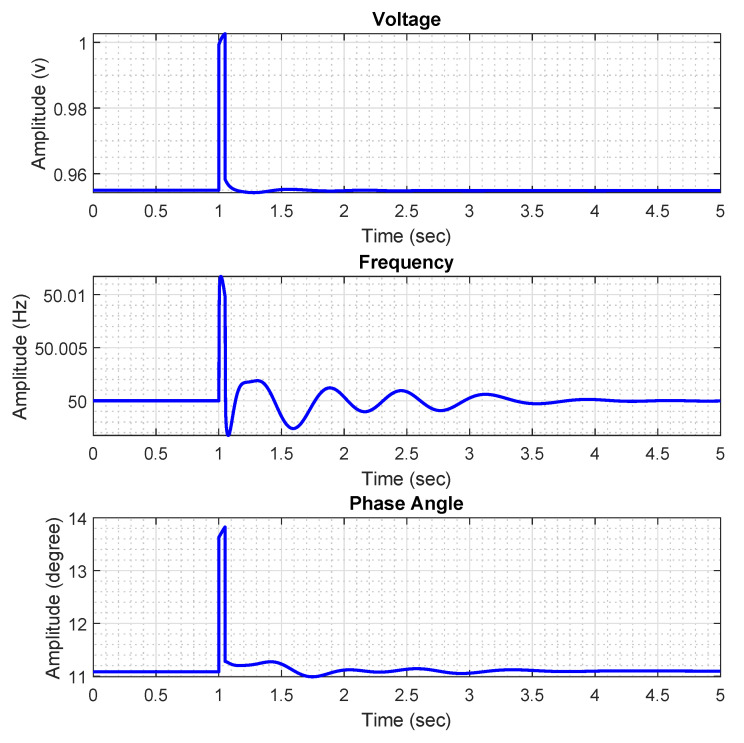
The collected voltage, frequency, and phase angle measurements following an LL fault on bus #1 at t=1 second of the simulation.

As mentioned earlier, we aim to diagnose different types of faults on the IEEE 118-bus system.This system contains 118 buses, 91 loads, and 19 generation buses. In this work, we simulate three different types of faults on this system. These faults are called load loss (LL), generator outage (G), and generator ground (GG). Together with the normal operational state of the system, there will be four types of states to be diagnosed. As for the simulation of the ‘LL’/‘G’ faults, we have disconnected the corresponding load/generation unit from its corresponding bus for a short period of time. As for the ‘GG’ faults, we have simulated a three-phase short-circuit fault between the generation units and ground. We have simulated 31 ‘LL’ faults by disconnecting each single of them from the corresponding bus. In the same way, 19 ‘G’ faults and 19 ‘GG’ faults are simulated. By adding the normal operational state of the system to the above-mentioned simulated faults, there exist 70 classes of operational states to be diagnosed. For each class or operational state, we have collected 500 samples from the sample that fault has been injected into either loads or generators, to the sample that fault has been cleared. Furthermore, voltage, frequency, and phase-angle features are collected from each bus of the system. In Figure 2, the voltage, frequency, and phase angle measurements collected from the first bus of the system in presence of an LL fault on bus #1 are illustrated. The fault has been injected at t=1 second and the simulation period is set to five seconds. As there are 118 buses in the system and three types of features are collected from each of them, there exist a total number of 354 features to be used in construction of datasets.

In order to study the effect of fault resistance (FR), signal to noise ratio (SNR), and sampling rate (SR) on the performance of the proposed diagnostic framework, 12 different datasets have been created. In this regard, two different SR values have been considered which are 20 KHz to 10 KHz. The FR values are supposed to be 1 Ω and 10 Ω, and the SNR values have been selected to be 50 dB, 40 dB, and 30 dB. By making a combination of the FR, SNR, and SR, 12 datasets $\left\{A_{1},\ldots ,A_{12}\right\}$ are generated.

Following the given description in Section 1, we consider two different scenarios and compare them with a baseline. Our baseline is the case, in which the raw data measurements are directly and without any processing fed into the classification models. Furthermore, in order to investigate the effectiveness of the proposed framework, we compare it with a scenario, in which the raw data measurements are firstly fed into the feature selection module, and, then, the selected features are directly fed into the classification module [6]. This is the first scenario (‘S#1’). In the second scenario, which is the proposed diagnostic framework in this study, we propose to generate the knockoffs of the selected features by means of the kGAN module, and, then, set these knockoffs as inputs to the classification models. Therefore, in the second scenario (‘S#2’), the raw data measurements are firstly fed into the feature selection module, where the selected features are further processed by the kGAN module and the generated knockoffs are fed into the classification models. As for the feature selection module, we resort to four well-known feature selection techniques including InfFS, MutInfFS, mRMR, and Relief. In the feature selection module, in order to find the best number of features to be selected, we start with two features and increase the number of features up to the value, for which no significant performance improvement can be observed for each classification model. The performance of each classification model has been reported based on the F-Measure.

**Figure 3:**
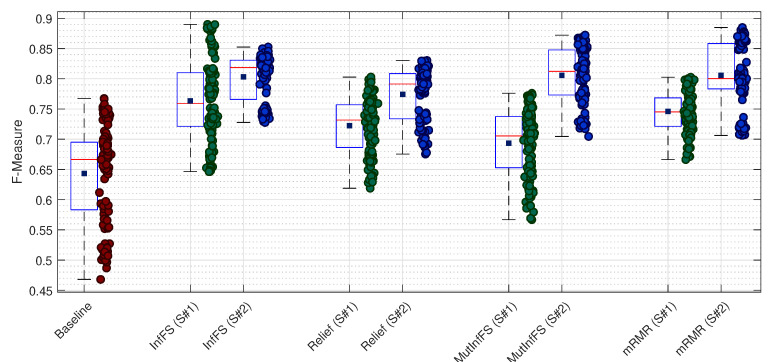
The attained F-Measure values for the kNN classification model w.r.t. each feature selection technique.

We start with the kNN classification model, where the attained results by means of this classifier are illustrated in Figure 3. It worth noting that each classification model is validated through a 10-fold cross-validation manner. As there are 12 datasets and a 10-fold cross-validation is performed, there are 120 F-Measure values for each experiment. In Figure 3, we have reported the results for all datasets for the baseline and the aforementioned feature selection techniques w.r.t. scenarios ‘S#1’ and ‘S#2’. As it can be observed from this figure, both scenarios have successfully improved the results compared with the baseline case. However, the second scenario (our proposed method) outperforms the first scenario [6] in all experiments, despite of the type of the feature selection technique. As for the first scenario, the attained results denote that InfFS leads to the highest average F-Measure value, which is then followed by mRMR, Relief, and MutInfFS. As for the second scenario, InfFS, MutInfFS, and mRMR show almost the same performance in terms of the average F-measure value, while Relief is the worst technique. Another worthwhile point to be mentioned is that the attained results for the second scenario show lower variation in the attained F-Measure values compared with the first scenario in the case of the InfFS, Relief, and MutInfFS, showing its robustness in dealing with different datasets. We have summarized the attained results of the kNN classification model in Table 1 w.r.t. each dataset in order to check for the effect of noise, fault resistance, and sampling rate on the performance of this classifier.

As it was mentioned earlier, in the first scenario, InfFS leads to the highest average F-Measure that is 0.7633, which is then followed by mRMR (0.7460), Relief (0.7224), and MutInfFS (0.6935). As for the second scenario, the best performance has been achieved by means of the mRMR (0.8057), which is then followed by MutInfFS (0.8055), InfFS (0.8030), and Relief (0.7743). In order to check the effect of FR on the performance of the proposed technique, we resort to the results of datasets $\left\{A_{1},\ldots ,A_{6}\right\}$ for which the FR is 1 Ω, and compare them with those of datasets $\left\{A_{7},\ldots ,A_{12}\right\}$, for which the FR is 10 Ω. For datasets with FR = 1 Ω, the average F-Measure for the second scenario is 0.8362, whereas it is 0.8331 for datasets with FR = 10 Ω. In the same vein, the average F-Measure for the first scenario are 0.7888 and 0.7718, respectively. Therefore, the attained results denote that the performance of the proposed method is not significantly affected by the value of FR. In order to check for the effect of noise, we regroup datasets into three groups of $\left\{A_{1},A_{4},A_{7},A_{10}\right\}$ with SNR = 50 dB, $\left\{A_{2},A_{5},A_{8},A_{11}\right\}$ with SNR = 40 dB, and $\left\{A_{3},A_{6},A_{9},A_{12}\right\}$ with SNR = 30 dB. For the second scenario, the average F-Measure values for the aforementioned three groups are 0.8291, 0.8190, and 0.7433, respectively. In the case of the first scenario, the attained results are 0.7762, 0.7247, and 0.6931, respectively. The attained results for both scenarios denote the superiority of the proposed method (the second scenario) in dealing with noisy data measurements compared with the first scenario. Finally, we aim to check for the effect of SR on the proposed method by regrouping the given datasets into two groups $\left\{A_{1},A_{2},A_{3},A_{7},A_{8},A_{9}\right\}$, for which the sampling rate is 10 KHz, and $\left\{A_{4},A_{5},A_{6},A_{1}0,A_{11},A_{12}\right\}$, for which the sampling rate is 20 KHz. The attained F-Measure values for the first scenario are 0.7167 and 0.7459 w.r.t. the aforementioned group of datasets, respectively. The average F-Measure values for the second scenario are 0.7958 and 0.7985, respectively. The attained results, on one hand, denote the superiority of the second scenario in comparison with the first scenario in dealing with datasets with different SR values. On the other hand, there is no significant changes for the second scenario when the SR decreases from 20 KHz to 10 KHz, denoting its robustness against the sampling rate issues.

**Figure 4:**
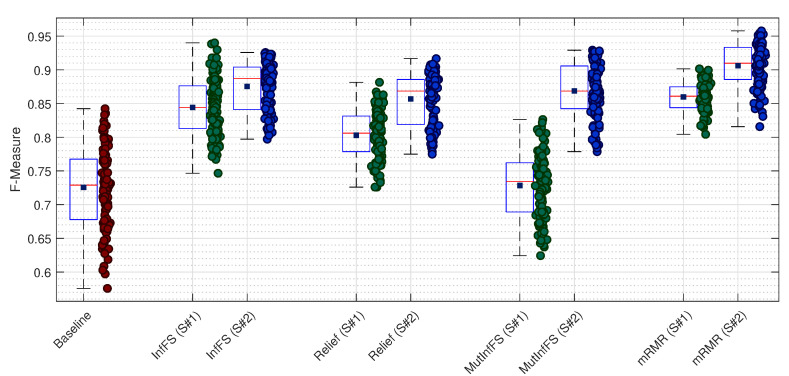
The attained F-measure values by means of the SVM classification model w.r.t. each dataset.

We repeat the same experiments for the SVM classification model. The attained results are represented in Figure 4. As it can be observed, the baseline shows much variation in terms of F-Measure in dealing with different datasets. However, it almost shows the same average F-Measure value in comparison with the results of the MutInfFS used in the first scenario, but lower average F-Measure values compared with the second scenario. By comparing the first and second scenarios, the attained results, on one hand, show the superiority of the second scenario for each feature selection technique. On the other hand, the second scenario leads to lower variation in F-Measure values when InfFS, Relief, and MutInfFS techniques are applied. The attained results for the SVM classification model are summarized in Table 2.

The collected results in Table 2 denote that mRMR leads to the best performance in both scenarios, which is then followed by InfFS, Relief, and MutInfFS in the first scenario, and InfFS, MutInfFS, and Relief in the second scenario. In order to investigate the effect of FR value, we regroup datasets as before, where the FR value is 1 Ω for the first group and 10 Ω for the second group. The average F-Measure value for the first group has been obtained as 0.8365 for the first scenario, whereas it is 0.7840 for the second group of datasets. In the same vein, the attained results for the second scenario are 0.8909 and 0.8627, respectively. The attained results of this experiment, on one hand, denote that the second scenario outperforms the first scenario. On the other hand, they verify the more robust performance of the second scenario compared with the first scenario against the changes in the FR value. In order to check for the effect of noise on the performance of the proposed scheme, same as what was done for the kNN classification model, we regroup datasets into three groups w.r.t. SNR = 50 dB, SNR = 40 dB, and SNR = 30 dB. For the first scenario, the average F-Measure values for each group of datasets can be computed as 0.8335, 0.8044, and 0.7928, respectively. As for the second scenario, the attained results are 0.9022, 0.8949, and 0.8331, respectively. As it can be observed from the attained average values of F-Measure for both scenarios, the second scenario outperforms the first one in dealing with noisy measurements. Finally, we regroup datasets into two groups based on the SR values, where SR = 10 KHz for the first group and SR = 20 KHz for the second group. The average F-Measure values for these two groups are 0.8048 and 0.8157, respectively, for the first scenario, whereas the average values for the second scenario are 0.8756 and 0.8779, respectively. The attained results verify the superiority of the second scenario over the first scenario.

Following the presented results for the kNN and SVM classification models, some general remarks can be made. Generally speaking, SVM classification model has outperformed the kNN by considering both scenarios plus the baseline, where the average F-Measure for the SVM is 0.8304, whereas it is 0.7508 for the kNN. The results of of the second scenario suggest that mRMR shows the best performance in dealing with the aforementioned datasets, however, its combination with the SVM classification model leads to a better combination for the sake of fault diagnosis in the IEEE 118-bus system. Furthermore, the results of both classification models verified the superiority of the proposed technique in comparison with the baseline and the first scenario.

The main advantage of the proposed diagnostic scheme is that it is data-driven, and, therefore, there is no need to have knowledge about the explicit model of the system. Further to this, we have proposed the use of kGAN module in order to generate a set of informative features from the selected measurements. This module can generate this set of features despite of the type of the distribution of data. Furthermore, the proposed diagnostic scheme can be easily extended to involve semi-supervised and unsupervised feature selection techniques in order to benefit from their advantages. The main drawback of the proposed framework goes back to the fact that this technique is offline and cannot be used for a real-time implementation.

## 5. Conclusions

This work is devoted to the design of a novel diagnostic framework for distributed power systems. The proposed diagnostic framework involves three modules including feature selection, kGAN, and decision making for the sake of fault diagnosis. It makes use of the voltage, frequency, and phase angle measurements collected by means of sparse measuring devices attached to each bus of the power system. The collected data measurements are firstly fed into the feature selection module in order to find the most informative features. The selected feature are then further processed by feeding them into the kGAN module, where a technique based on the GANs has been used in order to generate the corresponding set of knockoffs of the selected features. Generated knockoff are finally fed into the decision making module, where two different classification models are utilized to diagnose different types of faults. A very comprehensive comparative study has been provided in order to investigate the performance of the proposed method in dealing with noisy data measurements, datasets with high fault resistance values, and datasets with different sampling rate values. The attained results verify the applicability, effectiveness, and superiority of the proposed framework in comparison with a literature work. Verifying the results of this work for other large-scale power systems by making use of other state-of-the-art feature selection techniques and classification models could be investigated in a future work.

## Figures and Tables

**Table 1:** The attained F-Measure (FM) values by means of the kNN classification model w.r.t. each dataset.

Dataset	Baseline	InfFS		Relief		MutInfFS		mRMR	
		S#1	S#2	S#1	S#2	S#1	S#2	S#1	S#2
$A_{1}$	0.7379	0.8527	0.8312	0.7718	0.7963	0.7479	0.8560	0.7758	0.8647
$A_{2}$	0.6809	0.7847	0.8209	0.7386	0.7858	0.7111	0.8478	0.7468	0.8563
$A_{3}$	0.6519	0.7149	0.7966	0.7169	0.6973	0.6962	0.7968	0.7279	0.7818
$A_{4}$	0.7522	0.8828	0.8408	0.7928	0.8052	0.7695	0.8632	0.7956	0.8733
$A_{5}$	0.7006	0.8020	0.8305	0.7587	0.7887	0.7329	0.8511	0.7599	0.8639
$A_{6}$	0.6620	0.7418	0.7415	0.7388	0.7019	0.7113	0.7757	0.7432	0.7885
$A_{7}$	0.6691	0.7754	0.8244	0.7217	0.8149	0.6571	0.8155	0.7658	0.8046
$A_{8}$	0.5668	0.7274	0.8183	0.6705	0.8070	0.5905	0.8051	0.7157	0.7957
$A_{9}$	0.4973	0.6535	0.7406	0.6371	0.7331	0.6180	0.7236	0.6828	0.7141
$A_{10}$	0.6884	0.8125	0.8298	0.7544	0.8194	0.7515	0.8211	0.7910	0.8077
$A_{11}$	0.5925	0.7317	0.8181	0.6981	0.8106	0.6880	0.8084	0.7391	0.7956
$A_{12}$	0.5187	0.6799	0.7438	0.6691	0.7343	0.6485	0.7287	0.7090	0.7217
Avg.	0.6432	0.7633	0.8030	0.7224	0.7743	0.6935	0.8055	0.7460	0.8057

**Table 2:** The attained F-Measure (FM) values by means of the SVM classification model w.r.t. each dataset.

Dataset	Baseline	InfFS		Relief		MutInfFS		mRMR	
		S#1	S#2	S#1	S#2	S#1	S#2	S#1	S#2
$A_{1}$	0.7970	0.9143	0.9156	0.8258	0.8936	0.7776	0.9159	0.8696	0.9413
$A_{2}$	0.7407	0.8898	0.9060	0.8157	0.8877	0.7520	0.9061	0.8600	0.9344
$A_{3}$	0.6799	0.8768	0.8414	0.7998	0.8084	0.7351	0.8480	0.8516	0.8840
$A_{4}$	0.8265	0.8780	0.9091	0.8571	0.8973	0.8007	0.9195	0.8864	0.9407
$A_{5}$	0.7749	0.8527	0.9003	0.8330	0.8901	0.7671	0.9090	0.8701	0.9361
$A_{6}$	0.7211	0.8481	0.8418	0.8254	0.8219	0.7560	0.8456	0.8611	0.8867
$A_{7}$	0.7341	0.8259	0.8886	0.7893	0.8694	0.7159	0.8697	0.8546	0.9156
$A_{8}$	0.6706	0.7977	0.8839	0.7611	0.8640	0.6746	0.8867	0.8377	0.9050
$A_{9}$	0.6187	0.7856	0.8170	0.7518	0.8000	0.6549	0.8019	0.8259	0.8513
$A_{10}$	0.7697	0.8373	0.8934	0.8147	0.8766	0.7353	0.8742	0.8823	0.9157
$A_{11}$	0.7143	0.8206	0.8865	0.7839	0.8668	0.6954	0.8656	0.8595	0.9085
$A_{12}$	0.6611	0.8030	0.8221	0.7767	0.8054	0.6762	0.8000	0.8568	0.8543
Avg.	0.7257	0.8441	0.8755	0.8029	0.8569	0.7284	0.8685	0.8656	0.9061

## Data Availability

Not applicable.
